# The Application of Intermittent Intraoperative Neuromonitoring (I-IONM) and Continuous Intraoperative Neuromonitoring (C-IONM) During Thyroid Surgery: A Single-Center Study

**DOI:** 10.3390/jcm14082612

**Published:** 2025-04-10

**Authors:** Bruno Cirillo, Gioia Brachini, Giuseppe Cavallaro, Mariarita Tarallo, Cecilia Carlino, Giulia Duranti, Martina Zambon, Andrea Mingoli, Luigi Simonelli, Marco Bononi

**Affiliations:** 1 Department of Surgery, Sapienza University of Rome, 00161 Rome, Italy; bruno.cirillo@uniroma1.it (B.C.); gioia.brachini@uniroma1.it (G.B.); giuseppe.cavallaro@uniroma1.it (G.C.); ceciliacarlinomd@gmail.com (C.C.); giuliaduranti95@gmail.com (G.D.); marty.zambon@gmail.com (M.Z.); andrea.mingoli@uniroma1.it (A.M.); luigi.simonelli@policlinicoumberto1.it (L.S.); marco.bononi@uniroma1.it (M.B.); 2 Department of Emergency Surgery, Policlinico Umberto I, 00161 Rome, Italy; 3 Department of Medico-Surgical Sciences and Biotechnologies, Sapienza University of Rome, 00185 Rome, Italy

**Keywords:** intraoperative neuromonitoring (I-IONM), continuous intraoperative neuromonitoring (C-IONM), vocal cord paralysis (VCP), thyroidectomy, recurrent laryngeal nerve (RLN) palsy, nerve monitoring

## Abstract

**Background:** The application of intermittent intraoperative neuromonitoring (I-IONM) and continuous intraoperative neuromonitoring (C-IONM) has been widely accepted to improve surgical outcomes after thyroid surgery. This observational study aimed to evaluate the impact of vocal cord paralysis (VCP) in thyroid surgery conducted with I-IONM and C-IONM. **Materials and Methods:** From January 2018 to December 2022, 147 patients operated on with I-IONM and C-IONM for thyroid surgery were analyzed. Variations in the rates of the occurrence of temporary and permanent vocal cord paralysis between the two groups were compared. A *p*-value < 0.05 was considered statistically significant. **Results:** In total, 147 patients were eligible for inclusion in the study. Of these, 96 (65%) patients underwent thyroid surgery with I-IONM, 52 patients (35%) underwent surgery with C-IONM by a single surgeon. The percentage of temporary VCP was 4.1% (4 patients) in the I-IONM group; no patients had permanent VCP. In the C-IONM group, two patients (3.9%) had permanent vocal cord paralysis, and temporary vocal cord paralysis was observed in other two patients (3.9%), who recovered their nerve function after speech therapy. No statistically significant differences were found in the two groups. **Conclusions:** In our study, both I-IONM and C-IONM proved effective in predicting VCP, and no significant differences were observed between the two techniques in our series.

## 1. Introduction

**Figure 1:**
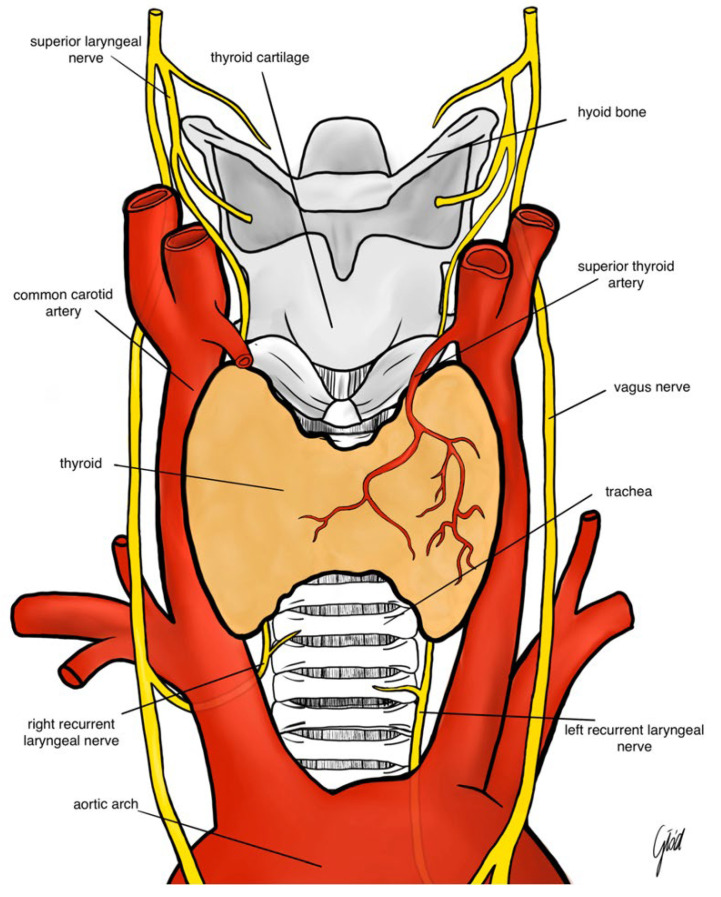
The anatomy of the recurrent laryngeal nerve and the superior laryngeal nerve.

Recurrent laryngeal nerve injury is the leading major complication of thyroid surgery. It decreases patients’ quality of life and exposes surgeons to medicolegal issues. The RLN can be damaged through several mechanisms, including stretching, crushing, thermal injury, electrical injury, and transection. Nerve injuries may be classified as neuropraxia, axonotmesis, and neurotmesis. Thyroid surgery is the etiology of 33% of iatrogenic unilateral vocal cord immobility and 80% of iatrogenic bilateral vocal fold immobility. The recurrent laryngeal nerve is more vulnerable to damage in the hands of less experienced surgeons in reoperative thyroid and neck surgery, for patients with large goiters or Graves’ disease, and in patients with a history of neck radiotherapy [1]. During thyroidectomy, recurrent laryngeal nerve (RLN) palsy and external branch of the superior laryngeal nerve (EBSLN) palsy are, next to hypoparathyroidism and postoperative bleeding, some of the most common complications. Identifying and preserving the RLN remains one of the most challenging aspects. The overall incidence of transient or permanent palsy after thyroid surgery is low (5–8 and 0.3–3 percent, respectively), but is associated with troubling consequences such as dysphonia, dyspnea during daily activities, dysphagia, and aspiration. Bilateral RLN injuries are less common than unilateral injuries, but if they occur, they can have grievous consequences for the patient regarding morbidity, such as compromising the functional integrity of the upper airways and consequently necessitating a tracheostomy. RLN is a branch of the vagus nerve. The left RLN is found to be inferior to the aortic arch and posterior to ligamentum arteriosum. The right vagus continues posteriorly to the apex of the right lung, giving rise to the right RLN which loops around the right subclavian artery. The recurrent laryngeal nerves then continue superiorly bilaterally and pass behind the lobe of the thyroid gland as they travel along the lateral surfaces of the trachea and esophagus in the tracheoesophageal groove. The nerves pass behind the cricothyroid joint as they enter the larynx at this level through fibers of the inferior constrictor muscles of the pharynx (Figure 1). The inferior branch of the recurrent laryngeal nerve innervates all the intrinsic muscles of the larynx, excluding the cricothyroid muscle (innervated by the superior laryngeal nerve). The recurrent laryngeal nerve also branches to the inferior constrictor and cricopharyngeal muscles before entering the larynx. RLN supplies the intrinsic muscle of the larynx, except the cricothyroid muscles. The RLN also carry general visceral sensory fibers from the region inferior to the glottis. Unilateral RLN injury causes damage to the vocal cords, as well as dysphonia and sometimes dysphagia. The incidence of one-sided palsy is 5–8% for transient damage and 1–2% for definitive damage. Bilateral RLN injuries can cause voice changes, and inspiratory dyspnea followed by laryngeal stridor, and some patients then need a tracheostomy. Traditionally, the gold standard for RLN preservation is its direct visualization. In the last 20 years, intraoperative nerve monitoring (IONM) has allowed the surgeon to better identify the nerve, especially when it is hard to visualize due to anatomical variation, reintervention, thyroid cancer, or recurrent goiter [2]. Neuromonitoring enables the identification of the site of the lesion and its etiology. Trauma-related injuries due to traction maneuvers are the most common, whereas incision or electrocoagulation are much less frequent mechanisms. There are two IONM techniques: intermittent and continuous. Intermittent intraoperative neuromonitoring (I-IONM) consists of stimulation with a probe by the surgeon during surgery to check the proper functioning of the RLN during its identification; visual identification of the nerve is always necessary. I-IONM is useful and easy to use, but a malfunction of the RLN is predicted only after nerve damage has occurred. Continuous IONM (C-IONM), on the other hand, through the placement of an Automatic Periodic Stimulation (APS^®^) electrode (Medtronic) on the vagus nerve (VN) and the continuous stimulation, gives a signal throughout the procedure, alerting the surgeon to any incorrect maneuvers, such as excessive traction, allowing them to anticipate the imminent lesion [3]. While I-IONM identifies the lesion only when it has already occurred, C-IONM enables the surgeon to correct and ideally avoid nerve damage and actual LOS. Both intermittent and continuous IONM allow for identification between segmental loss of signal (LOS type 1) and diffuse (LOS type 2); this facilitates the instant detection and release of a distressed nerve and can minimize RLN injury [4] or even change intraoperative decisions. In this study, we aimed to evaluate the incidence rate of vocal cord paralysis (VCP) in thyroid surgeries performed with C-IONM or I-IONM.

## 2. Methods

A retrospective cohort study was performed to compare thyroid surgical patients undergoing thyroidectomy with I-IONM or C-IONM by a single surgeon at the Policlinico Umberto I—Sapienza University of Rome. The study period was January 2018–December 2022. Data on demographics, types of thyroid disease (nontoxic multinodular goiter [NMG], toxic multinodular goiter [TMG], thyroiditis, thyroid carcinoma, atypical adenoma), types of surgical intervention (lobectomy, total thyroidectomy), operative time, and the pathological examination of the specimen were collected. Particular attention was given to evaluating vocal cord paralysis (VCP), whether temporary or permanent, and the type of IONM used. The exclusion criteria were patients undergoing secondary surgery. All patients provided written informed consent and the study was approved by the department board.

### 2.1. Technique

All patients underwent a fibrolaryngoscopic examination of the vocal cords preoperatively. Working together with the anesthesiologist has a fundamental role in performing thyroid surgery using neuromonitoring of the laryngeal nerves. All the patients underwent general anesthesia and were intubated with an appropriately selected endotracheal tube, ensuring that the surface electrodes adhered to the true vocal cords. No muscle relaxants were used.

A Nerve Integrity Monitor (NIM-Response) and electromyography (EMG) endotracheal tube (Medtronic, Jacksonville, FL, USA) were used for monitoring. During the surgical procedure, IONM, using either intermittent or continuous techniques on the basis of the availability of the equipment, was applied by the surgeon together with the direct visualization of the RLN, which is always performed.

After surgery, if there was a loss of signal or paresis of the vocal cords, dysphonia, dysphagia, or other clinical symptoms, the patients underwent postoperative fibrolaryngoscopy, the first as soon as possible after surgery, and then at 2, 4, and 6 months after surgery. A paralysis of the RLN was defined as definitive if there was no recovery of vocal cord function after 6 months.

### 2.2. Neuromonitoring

#### 2.2.1. I-IONM

Intermittent recurrent laryngeal nerve monitoring consists of electrostimulations of the RLN after its visualization, recording to electrodes placed on the surface of the endotracheal tube near the vocal cords. First described in 1966 by Shedd DP et al. [5] in experimental studies conducted on dogs and then in two human subjects. He shows that the stimulation (via a probe, with a 1 mA pulse) of RLN can be detected by an endotracheal balloon placed in the larynx. Several studies propose standardization of IONM. The International Neural Monitoring Study Group has proposed a formula following a sequence of steps [6,7]. It consists of the evaluation of vocal function on laryngoscopy before the intervention, the evaluation of the integrity of the Vagal Nerve (VN) and RLN before and after the dissection, and the evaluation of vocal functions after surgery [6]. I-IONM devices without EMG, only with an acoustic signal, do not allow a clear distinction between artifacts and potential vocal cord distress [8]. The I-IONM technique is useful in thyroid surgery in three main areas: support to the identification of RLN, repeated stimulation of the surrounding tissues, and nerve itself allowing for the location of the different courses of the nerve in the operating field, especially in cases of modified anatomy.

#### 2.2.2. C-IONM

Continuous recurrent laryngeal nerve monitoring differs from I-IONM in the use of an electrode (APS) placed on VN, beyond the EMG display, with the endotracheal tube with electrodes on its surface and the probe for intermittent stimulation. This allows for a constant evaluation of RLN function predicting imminent lesion. First described by Lamade et al. in 1997 [9,10], it was then overhauled by the same author because the first system described using a double-balloon endotracheal tube with stimulation was not suitable for routine surgery. There are two main types of C-IONM to monitor neural activity during surgery: the CMAP-based methodology (Compound muscle action potentials—CMAP—in laryngeal muscles), which uses a vagal nerve electrode, and the LAR-based methodology, described by Sinclair et al., which relies on the Laryngeal Adductor Reflex (LAR), triggered by supraglottic laryngeal mucosal stimulation [11]. We adopted the CMAP-based methodology. The same scheme proposed by the International Neural Monitoring Study Group [6] can be applied to the C-IONM by a preoperative evaluation of the vocal cord function and a stimulation of VN to evaluate its integrity before placing the electrode. C-IONM gives real-time information on the status of the nerves, and it can also help to identify intraoperatively the recovery of the lesion and predict postoperative complications [12]. The C-IONM technique has the potential of preventing unilateral RLN injury, which is not the case for the IONM technique.

### 2.3. Loss of the Neuromonitoring Signal (LOS)

Generally, a baseline amplitude of >500 µV was considered assessable. Even if the baseline signal had been lower than 500, we would have still considered it valid, as all patients had undergone preoperative fibrolaryngoscopy, confirming proper bilateral vocal cord function.

Loss of neuromonitoring signal (LOS) was defined as a signal drop of nerve amplitude to less than 100 µV. Two types of LOS have been described [13]:

- LOS type 1, segmental: the signal cannot be confirmed near a specific point of RLN during stimulation, but is distal to the point of injury;
- LOS type 2, global: there is no response to stimulation over VN and RLN.

C-IONM helps to evaluate the recovery of the nerve intraoperatively. An enhancement of >100 µV but less than 50% of the baseline reflect an incomplete recovery; when the amplitude rehabilitates with a value more than 50% of the baseline, there is a complete recovery. A decrease in amplitude > 50 with an increase in latency > 10% is called “combined events” and it is suggestive of traction trauma and can be followed simultaneously by C-IONM. Combined events can be reversible in 80% of cases when the traction is terminated. If the recovery is incomplete within 20 min, both type 1 (95%) and type 2 (70%) lead to a postoperative VPC [14]. The neuromonitoring helps to identify the mechanism of injury.

The most common cause of signal loss was traction trauma [13]. The most frequent injuries following traction are thermal damage, compression, clamping, ligature, entrapment, and transection. Among these, thermal injury, clamping, and traction are the most severe, posing the highest risk for long-term VCP. Specifically, the risk percentages are 28% for thermal injury, 50% for clamping, and 1.4% for traction [15,16].

During a thyroidectomy, if a loss of signal (LOS) occurs during neuromonitoring, several maneuvers can be performed to attempt signal restoration. For C-IONM, it is critical to verify the correct placement of the APS electrode and ensure the endotracheal tube has not been displaced during the procedure. If the LOS is related to the RLN, direct stimulation of the nerve using a probe with a current of 1–2 mA can be employed to attempt to elicit a response. This intermittent stimulation, used in both I-IONM and C-IONM, is performed by stimulating the RLN from its laryngeal entry point to the vagus nerve. This helps determine whether the LOS is type 1 or type 2 and identifies the point of damage in cases of type 1 LOS. Particular care must be taken to assess the surgical context and consider the possibility that the signal loss may be temporary, especially if it occurs during phases where the nerve is under transient stress, such as manipulation or exposure. Once the cause of the signal loss has been identified and addressed, it is essential to continue monitoring the nerve throughout the remainder of the surgery to prevent further complications. The anesthetist may administer corticosteroids to minimize inflammatory or irritative factors and assess potential intraoperative signal recovery. If the signal is restored, postoperative nerve monitoring is equally crucial to track recovery and ensure that no permanent vocal cord paralysis occurs.

### 2.4. Statistical Analysis

To identify any significant relation between the considered variables and the procedures, the following tests were performed:

The *t*-test was used for quantitative variables, while for categorical variables, the Fisher exact test and the chi-squared test were employed.

A *p*-value of 0.05 was taken as reference for statistical significance. All the statistics with a *p*-value lower than 0.05 are considered significant.

## 3. Results

A total of 147 patients were eligible for inclusion in the study. Of these, 96 (65%) patients underwent thyroid surgery with I-IONM. In this group, 60 (62.5%) patients underwent surgery for nontoxic multinodular goiter, 12 (12.5%) patients for carcinoma. No patient had permanent vocal cord paralysis (VCP); four (4.1%) patients had temporary VCP. Mean age was 57.65 years, and 71 (74%) patients were female. In the second group, 52 patients (35%) underwent surgery with C-IONM, the mean age was 58 years, and 36 (69.2%) patients were female. A total of 13 (25.5%) patients were affected by thyroid carcinoma. A total of four patients (7.8%) had VCP in total; in two cases, temporary VCP was observed, who recovered their nerve function after speech therapy. Toxic multinodular goiter accounted for the largest proportion in the I-IONM group, accounting for 14.5% compared to the C-IONM group percentage (5.9%). Regarding the surgical technique, a bilateral lobectomy was performed on 66 patients (68.7%) in the I-IONM group, and on 36 patients in the C-IONM group (70.6%). No significant difference was found regarding age (*p* = 0.8892), sex (*p* = 0.8085), operative time (*p* = 0.0707), and total vocal cord paralysis (*p* = 0.7386) (Table 1).

## 4. Discussion

Thyroid surgery is globally a high-volume surgery, with approximately 150,000 thyroidectomies performed per year in the U.S. A recurrent laryngeal nerve (RLN) or external branches of superior laryngeal nerve injury remains the most fearsome complication of thyroid surgery. VCP caused by recurrent laryngeal nerve injury is a common postoperative complication in thyroid surgery. Several studies have demonstrated the effectiveness of the I-IONM in identifying and preserving the integrity of the RLN in thyroid surgery, to prevent postoperative paralysis. I-IONM also allows for the real-time monitoring of target nerve integrity and an evaluation of the nerve injury mechanism. The introduction and the development of the intraoperative neuromonitoring I-IONM and C-IONM allow for a better identification of the RLN when it is hard to visualize due to anatomical variation, fibrosis, thyroid cancer, recurrent goiter, and reoperation. I-IONM enables the surgeon to evaluate the integrity and the function of the nerve after its dissection reducing the risk of bilateral palsy. Maowei Pei et al. [17], in a study of 109 patients, compare the I-IONM with visualization of the RLN alone in thyroid reoperations. They found that in the I-IONM group, the incidence of RLN injury was 5.3% against 13.8% in the visualization group. The incidence of surgeon-related RLN injury was 0% in the I-IONM group and 7.7% in the visualization-alone group (VA). In a meta-analysis, Pisanu et al. [18] found no differences between the I-IONM group and the VA group, finding the same rates of transient and permanent palsy. Other studies show that I-IONM decreases only in transient RLN injury [19,20] or only in permanent ones [21].

Intermittent neuromonitoring has some limitations. It only gives information at the time of stimulation, so the period between two stimulations is a critical time for RLN injuries, and any damage can only be detected after it has occurred. I-IONM informs the user about a function of distal part of the nerve and cannot determinate if there is a proximal lesion at the point of stimulation [14,22]. This limitation is made obsolete by the use of C-IONM, which can predict imminent lesions by the continuous neuromonitoring of the vagus-RLN axis. Some studies compare I-IONM and C-IONM, finding the last one to be superior in preventing VCP and predicting postoperative complications; Schneider et al. found that with C-IONM, early and permanent VCP were, respectively, 1.7 and 30 times lower than I-IONM [23]. Continuous neuromonitoring has better reliability than an intermittent one. I-IONM has a sensitivity of 63.0–91.3% and a specificity of 97.1–99.5%, while C-IONM has a sensitivity of 90.9–100% and a specificity of 90.2–99.7%. It also has a lower rate of false positives and false negatives [24]. The predictive accuracy of continuous neuromonitoring helps surgeons in intraoperative decision; for example, in case of multinodular goiter or bilateral surgery for benign pathology, when there is a loss of signal for one side, the surgeon can consider a second surgery for the contralateral part to safeguard at least one RLN [8]. Even C-IONM has some limitations: first, it requires the preparation of VN, then it takes time to learn how to use it, but its use is possible even with C-IONM palsy of RLN, because it helps for constant stress and not for acute injuries such transection or clamping [12]. In the literature, different studies report the safety of both I-IONM and C-IONM, evaluating their applicability in pediatric patients and high-risk population as well [25]. However, there are few cases reported of complications related to VN monitoring, such as the temporary paralysis of VN and hemodynamic instability [26].

In our study, we compared the two techniques in terms of protecting the recurrent laryngeal nerve during thyroid surgery. According to the results, no statistical difference was shown in both groups in postoperative VCP, in accordance with the evidence from the current literature. The incidence of temporary VCP in the I-IONM group was 4.1%, while in C-IONM, it was 3.9%. The incidence of permanent VCP in the I-IONM group was 0%, lower than the C-IONM group (3.9%), without statistically significative differences (*p*-value 0.1). In the group of patients operated on with C-IONM, a higher incidence of carcinoma was observed (approximately twice as high, 25.5% vs. 12.5%). This could explain the difference in VCP rates, particularly regarding the two permanent cases. Numerous studies in the literature report similar findings, and specific guidelines have been established for managing the nerve in cases of neoplastic pathology [27].

This study had several limitations. Firstly, as this is a retrospective study, we were unable to establish proper inclusion criteria to ensure balanced patient selection and group distribution; secondly, the sample size was moderately small, but it is limited because is a single surgeon cohort. Lastly, the lack of data on follow-up is also a limitation.

## 5. Conclusions

Nerve monitoring during thyroid surgery is a challenging process that includes three interdependent steps of recurrent laryngeal nerve evaluation: preoperative, intraoperative, and postoperative assessments of RLN function. The application of IONM during thyroid surgery has been extensively utilized to avoid the injury of the nerve. In conclusion, in our study, both techniques are effective in predicting VCP and there are no differences between I-IONM and C-IONM in our series. Future prospective studies would be useful.

## Figures and Tables

**Table 1:** Comparison of Patient Characteristics and Outcomes Between I-IONM and C-IONM Thyroid Surgery Groups.

Variabile	I-IONM	C-IONM	p-Value
Patients	96	51	
Age *	57.65	58	0.8892
Sex (M:F) ^	25:71	15:36	0.8085
Thyroid disease			
NMG ^	60 (62.5)	25 (49.1)	0.1615
TMG °	14 (14.5)	3 (5.9)	0.1749
Thyroiditis °	7 (7.3)	5 (9.8)	0.7528
Thyroid carcinoma ^	12 (12.5)	13 (25.5)	0.0776
Atypical adenoma °	3 (3.1)	5 (9.8)	0.1262
Bilateral lobectomy ^	66 (68.7)	36 (70.6)	0.9633
Unilateral lobectomy ^	14 (14.5)	7 (13.7)	1
Operative time (minutes) *	140.6771	156.8627	0.0707
Temporary VCP °	4 (4.1)	2 (3.9)	0.1828
Permanent VCP °	0 (0)	2 (3.9)	0.1134
Total of VCP°	4 (4.1)	4 (7.8)	0.7386

* = *t*-test, ^ = Chi-square test, ° = Fisher’s exact test, NMG = nontoxic multinodular goiter, TMG = toxic multinodular goiter.

## Data Availability

Data are available upon request with the permission of the last author.
